# Adiponectin Attenuates Angiotensin II-Induced Vascular Smooth Muscle Cell Remodeling through Nitric Oxide and the RhoA/ROCK Pathway

**DOI:** 10.3389/fphar.2016.00086

**Published:** 2016-04-07

**Authors:** Wared Nour-Eldine, Crystal M. Ghantous, Kazem Zibara, Leila Dib, Hawraa Issaa, Hana A. Itani, Nabil El-Zein, Asad Zeidan

**Affiliations:** ^1^Cardiovascular Physiology Lab, Department of Anatomy, Cell Biology and Physiology, Faculty of Medicine, American University of BeirutBeirut, Lebanon; ^2^ER045, Laboratory of Stem Cells, Department of Biology, Faculty of Sciences, The Lebanese UniversityBeirut, Lebanon; ^3^Division of Clinical Pharmacology, Department of Medicine, Vanderbilt University School of Medicine, NashvilleTN, USA

**Keywords:** adiponectin, angiotensin II, nitric oxide, VSMC, remodeling

## Abstract

**Introduction:** Adiponectin (APN), an adipocytokine, exerts protective effects on cardiac remodeling, while angiotensin II (Ang II) induces hypertension and vascular remodeling. The potential protective role of APN on the vasculature during hypertension has not been fully elucidated yet. Here, we evaluate the molecular mechanisms of the protective role of APN in the physiological response of the vascular wall to Ang II.

**Methods and Results:** Rat aortic tissues were used to investigate the effect of APN on Ang II-induced vascular remodeling and hypertrophy. We investigated whether nitric oxide (NO), the RhoA/ROCK pathway, actin cytoskeleton remodeling, and reactive oxygen species (ROS) mediate the anti-hypertrophic effect of APN. Ang II-induced protein synthesis was attenuated by pre-treatment with APN, NO donor *S*-nitroso-*N*-acetylpenicillamine (SNAP), or cGMP. The hypertrophic response to Ang II was associated with a significant increase in RhoA activation and vascular force production, which were prevented by APN and SNAP. NO was also associated with inhibition of Ang II-induced phosphorylation of cofilin. In addition, immunohistochemistry revealed that 24 h Ang II treatment increased the F- to G-actin ratio, an effect that was inhibited by SNAP. Ang II-induced ROS formation and upregulation of p22^phox^ mRNA expression were inhibited by APN and NO. Both compounds failed to inhibit Nox1 and p47^phox^ expression.

**Conclusion:** Our results suggest that the anti-hypertrophic effects of APN are due, in part, to NO-dependent inhibition of the RhoA/ROCK pathway and ROS formation.

## Introduction

Hypertension is a primary risk factor for other cardiovascular diseases, such as myocardial infarction, cardiac hypertrophy, heart failure, atherosclerosis, and vascular hypertrophy (Zeidan et al., 2003; Zhang et al., 2005; Pedrinelli et al., 2012). These diseases are governed by structural and functional changes in the cardiovascular system. Angiotensin II (Ang II) is a potent vasoconstrictor that binds to its angiotensin receptor (AT_1_) in different organ systems to increase blood pressure. In the vascular system, Ang II binds to AT_1_ receptors to induce vascular smooth muscle cell (VSMC) contraction and subsequent vasoconstriction (Bagi et al., 2011). Moreover, Ang II promotes VSMC remodeling, cell growth, fibrosis, collagen deposition, and contractility (Touyz, 2005; Heeneman et al., 2007).

The molecular mechanisms associated with VSMC growth and contraction include reactive oxygen species (ROS) formation and the activation of several signaling pathways, such as MAPK and the RhoA/ROCK pathway (Zeidan et al., 2007). The RhoA/ROCK pathway is activated by several hypertension-associated factors, such as the Ang II (Carbone et al., 2015), endothelin-1 (Et-1; Homma et al., 2007), leptin (Zeidan et al., 2007), and mechanical stretch (Zeidan et al., 2003). Activation of the RhoA/ROCK pathway leads to the phosphorylation and subsequent inhibition of the actin-depolymerization enzyme cofilin, and thus promotes formation of stress fibers, an increase in F-actin formation, and depletion in G-actin (Zeidan et al., 2007). Hence, the RhoA/ROCK pathway affects cell morphology, produces modifications in actin cytoskeleton, and regulates transcription factors leading to cellular hypertrophy (Zeidan et al., 2006).

Nitric oxide (NO) exerts a protective role on cardiovascular function (Jones and Bolli, 2006). Activation of endothelial nitric oxide synthase (eNOS) produces NO, which exerts vasoprotective effects on the vascular wall by maintaining vasodilation, anti-coagulation, and anti-oxidation (Zhou et al., 2004). Moreover, NO primarily mediates endothelium-dependent relaxation through activation of soluble guanylyl cyclase, leading to an increased production of cGMP which, in turn, activates several phosphodiesterases and cGMP-dependent protein kinases (Mayer et al., 1998). However, the precise mechanisms by which NO prevents vascular pathology are not well understood.

Evidence suggests that the hypertrophic signaling pathways triggered in response to Ang II are mediated by ROS formation (Dai et al., 2011). The enhanced production of ROS increases free radicles and leads to oxidative stress, which in turn induces vascular dysfunction and remodeling (Touyz and Briones, 2011; Montezano and Touyz, 2012). ROS is produced by a multi-subunit complex protein called NADPH oxidase, which consists of several membrane-bound proteins/components including Nox1, Nox4, p22^phox^ and cytosolic components including Rac1 and p47^phox^. Many studies have shown the important role of p22^phox^ and p47^phox^ in Nox1 activation in VSMC (Ushio-Fukai et al., 1996; Lavigne et al., 2001; Van Heerebeek et al., 2002). Moreover, studies have shown the involvement of p22^phox^ in different agonists-induced ROS formation, including Ang II (Ushio-Fukai et al., 1996).

Adiponectin (APN) is a circulating adipokine that plays a protective role in the cardiovascular system (Caselli et al., 2014). It exerts potent anti-atherosclerotic, anti-inflammatory, and anti-diabetic effects (Shibata et al., 2005; Tajtakova et al., 2010). Plasma APN levels negatively correlate with cardiovascular disease, such as hypertension and metabolic disorders (Zhu et al., 2008). Physiological concentrations of APN range between 5 and 25 μg/mL (Ouchi et al., 1999) and are significantly reduced in hypertensive patients (Kim et al., 2013). This reduction in adiponectin is believed to be responsible for hypertension-associated cardiovascular diseases (Menzaghi et al., 2007; Kawai et al., 2013). Several studies have investigated the relationship between Ang II and adiponectin (Uchida et al., 2008; Ohashi et al., 2011). It has been shown that adiponectin has the ability to protect against Ang II-induced cardiac fibrosis (Fujita et al., 2008) and prevent Ang II-induced apoptosis in human endothelial cells (Lin et al., 2004). Furthermore, APN expression ameliorates the development of hypertension in APN-deficient mice and hypertensive KKAy mice (Ohashi et al., 2006). Uchida et al. (2008) have shown that Ang II infusion decreased plasma adiponectin levels via angiotensin receptor-1. Moreover, renin angiotensin system inhibition was associated with increased plasma adiponectin levels (Uchida et al., 2008).

Adiponectin is secreted predominantly by adipose tissue (reviewed in Ghantous et al., 2015a), but several studies have shown the ability of different tissues to produce APN (reviewed in Ghantous et al., 2015a). Although, many investigations have tried to uncover the molecular mechanisms responsible for the cardioprotective effects of APN, the various signaling mechanisms in VSMC remain unclear. In this study, we examined whether APN exerts protective effects against Ang II-induced vascular hypertrophy. Moreover, we investigated the possible role of NO, ROS, RhoA/ROCK pathway, and actin cytoskeleton dynamics.

## Materials and Methods

### Rat Aorta Organ Culture

Male Sprague-Dawley rats (200–250 g) were killed by CO_2_, as approved by the Animal Ethics Committee, American University of Beirut. The thoracic aorta was isolated and stripped of the surrounding adipose and connective tissue using *N*-HEPES buffer solution (400 mmol/L NaCl, 200 mmol/L KCl, 100 mmol/L MgCl_2_, 100 mmol/L HEPES, 11.5 mmol/L glucose) in 5% penicillin-streptomycin. Aortic rings were then transferred to DMEM/F-12 HAM culture media and incubated at 37°C, 5% CO_2_. Different inhibitors and agonists were added to the media 1 h before adding Ang II (1 μmol/L; Sigma-Aldrich, St. Louis, MO, USA) as follows: actin depolymerizing agent Cytochalasin D (Cyt-D, 1 μmol/L, Sigma-Aldrich, St. Louis, MO, USA), the NO donor *S*-nitroso-*N*-acetylpenicillamine (SNAP, 0.1 mmol/L, Sigma-Aldrich, St. Louis, MO, USA), the cGMP analog 8-Bromo-cGMP (cGMP, 100 μmol/L, Santa Cruz Biotechnology, Santa Cruz, CA, USA), the specific inhibitor of cGMP-dependent protein kinase Rp-8-Br-PET-cGMPS (cGMPS, 50 nmol/L, Santa Cruz Biotechnology, Santa Cruz, CA, USA), adiponectin (5 μg/ml, Santa Cruz Biotechnology, Santa Cruz, CA, USA), and NG-nitro-L-arginine methyl ester (L-NAME; 2 mmol/L, Sigma-Aldrich, St. Louis, MO, USA). The concentrations of Ang II and all pre-treatments used in this study were determined in preliminary studies to avoid cytotoxicity while still maintaining effectiveness. Following incubation, aortic rings were SNAP-frozen in liquid nitrogen and stored.

### Measurement of Leucine Incorporation

Protein synthesis was examined by analysis of [^3^H]-leucine incorporation. Endothelium-intact and denuded aortic rings were cultured with or without inhibitors for 24 h and with [^3^H]-leucine in order to measure protein synthesis as described previously (Zeidan et al., 2003).

### Vascular Function Studies

The thoracic aorta rings were placed immediately in ice-cold HEPES-buffer solution. The composition of the HEPES-buffer solution was in mmol/L: NaCl 135.5, KCl 5.9, CaCl_2_ 2.5, MgCl_2_ 1.2, glucose 11.6, HEPES 11.6, pH = 7.4. High-K^+^ solution (60 mmol/L) was obtained by isomolar exchange of NaCl for KCl. Aortic rings were cultured with/without Ang II (1 μmol/L) for 24 h and pre-treated with/without adiponectin (5 μg/ml), the NO donor SNAP (0.1 mmol/L), 8-Br-cGMP (cGMP; 100 μmol/L), the specific inhibitor of cGMP-dependent protein kinase Rp-8-Br-PET-cGMPS (cGMPS, 50 nmol/L), or L-NAME (2 mmol/L). After that, cultured rat aortic segments were washed and mounted in a myograph (620 M, Danish Myo Technology, Aarhus, Denmark) and equilibrated at a resting tension of 2 g for 45 min. The resting tension was readjusted every 15 min during equilibration time before starting the experiments. After resting tension of each aortic ring have stabilized, maximum isometric contraction was measured after addition of high-K^+^. After that, the aortic rings were pre-contracted with 1 μmol/L phenylephrine (PE). When the plateau was reached, a single concentration of 1 μmol/L acetylcholine (ACh) was added to evaluate endothelium functional integrity of the aortic rings (Furchgott, 1983). The aortic ring was discarded if relaxation with ACh was equal to or lower than 80% of the maximal contraction obtained in aortic rings pre-contracted with PE. The inability of 1 μM ACh to induce muscle relaxation in the denuded aortic ring indicated the removal of endothelial cells. Contractile responses to 1 μmol/L PE and subsequent relaxing responses to 0.1 mmol/L sodium nitroprusside were also compared in aortic ring with or without endothelium to ensure that the VSMC was not damaged during removal of the endothelium. Results in isometric contractions were recorded using 620 M myograph connected to a PowerLab 4/35 data acquisition system (AD Instruments, Sydney, NSW, Australia) and saved in a computer. Results were expressed as mN/mm.

### Protein Extraction and Western Blotting

Treated aortic rings were transferred to liquid nitrogen and homogenized in order to extract proteins. The rings were added to 300 μL lysis buffer (50 mmol/L Tris, pH = 8.0, 150 mmol/L NaCl, 1% Nonidet-P40, 0.5% sodium deoxycholate) with protease inhibitors. They were centrifuged at 9,000 rpm for 5 min at 4°C. The supernatant protein solution was then aspirated and quantified using Bradford assay. Equal amounts of extracted proteins were then loaded on a 12% SDS-PAGE and transferred to a nitrocellulose membrane. Blots were then probed with specific antibodies, at 1:1000 dilution in 5% BSA, against RhoA, p-cofilin, and actin (Sigma-Aldrich, St. Louis, MO, USA). Proteins were detected using ECL immunoblotting detection system (Santa Cruz Biotechnology, Santa Cruz, CA, USA). The Western blot images were acquired using a ChemiDoc MP System and Image Lab software, followed by quantification of the blots using ImageJ software.

### RNA Isolation and Real-Time PCR

Ribonucleic acid (RNA) was isolated from control or treated aortic rings and Real-Time PCR was done as previously described. The primers were: Nox1 forward 5′-TTTCCTAAACTACCGACTC-3′ and Nox1 reverse 5′-GTGCGACAACGGACTATC-3′, p22^phox^ forward 5′-TGGCCTGATCCTCATCACAG-3′ and p22^phox^ reverse 5′-AGGCACGGACAGCAGTAAGT-3′, p47^phox^ forward 5′-GCTGCCCACACCTCTTGAACTT-3′ and p47^phox^ reverse 5′-TCTTCAGGCAAAACCACCA-3′, and 18S rRNA forward 5′-GTAACCCGTTGAACCCCATT-3′ and 18S rRNA reverse 5′-CCATCCAATCGGTAGTAGCG-3′ which was used as the housekeeping gene to normalize expression.

### Analysis of RhoA Activity

To assess the effect of Ang II on RhoA activity in aortic rings, we measured the level of RhoA–GTP using the RhoA G-LISA Activation Assay Kit as per the manufacturer’s instructions (Cytoskeleton, Denver, CO, USA).

### Measurement of RhoA Translocation by Western Blot

Tissue lysates obtained after homogenization were collected in a homogenization buffer (pH = 7.5) containing 2 mmol/L Tris, 1 mmol/L DTT, 2 mmol/L EDTA, 2 mmol/L EGTA, 50 mmol/L NaF, 0.2 mmol/L Na_3_VO_4_ and protease inhibitor cocktail. The homogenate was then sheared using a 26G needle and pelleted by a 10 min centrifugation at 5,000 rpm at 4°C. The supernatant was further centrifuged at 57,000 rpm for 60 min at 4°C and the resulting supernatant was the cytosolic fraction. The pellet was re-homogenized in lysis buffer (pH = 7.5) containing 50 mmol/L Tris, 150 mmol/L NaCl, 2 mmol/L EDTA, 2 mmol/L EGTA, 50 mmol/L NaF, 0.2 mmol/L Na_3_VO_4_, 10 mmol/L Na4PO7, 40 mmol/L ß-glycero-phosphate, 1% Triton X-100, 10% glycerol, and protease inhibitor cocktail. The resulting homogenate was the membrane fraction. Both fractions, cytosolic (12 μg) and membrane (25 μg) were then blotted with anti-RhoA antibody (Santa Cruz Biotechnology, Santa Cruz, CA, USA), as previously explained (Zeidan et al., 2007).

### Measurement of F/G Actin Ratio by Western Blot

Aortic rings were homogenized and added to lysis buffer (50 mmol/L PIPES, 50 mmol/L NaCl, 5 mmol/L MgCl_2_, 5 mmol/L EGTA, 5% glycerol, 0.1% Nonidet-P40, 0.1% Triton X-100, 0.1% Tween 20, 0.1% β-mercaptoethanol, 1 mmol/L ATP, 1:100 protease inhibitor). The homogenate was then sheared using a 26G needle and centrifuged at 2,000 rpm for 5 min at 37°C. The resulting supernatant was removed and then ultra-centrifuged at 100,000 *g* for 1 h at 37°C in order to separate the F-actin from the G-actin, present in the pellet and the supernatant respectively. The supernatant was removed and ready to use, while the pellet was re-suspended using Cytochalasin D (10 μmol/L) which depolymerizes F-actin into G-actin. The solution was then incubated on ice for 1 h and suspended up and down every 15 min. After the addition of Laemmli, resulting G- and F-actin samples were denatured by heat then loaded on a 12% acrylamide gel and the membrane blotted with anti-actin antibody (Cell Signaling Technology, Danvers, MA, USA).

### Immunohistochemistry of RhoA Translocation

Frozen aorta tissue sections were fixed in 4% paraformaldehyde for 15 min at room temperature, then rinsed twice with PBS, and permeabilized with 0.2% Triton X-100 for 20 min. Blocking was done for 1 h with a blocking solution consisting of 1% BSA and 0.1% Triton X-100 in PBS. Sections were then incubated overnight with anti-RhoA primary antibody at 1:100 dilution in 1% BSA and 0.05% Tween-20, then rinsed twice with 0.1% Tween-20. A goat anti-rabbit secondary antibody, conjugated to Alexa Fluor (AF594 IgG, Invitrogen, USA), was then added at 1:250 dilution in 1% BSA and 0.05% Tween-20 for 1 h in the dark. Slides were then rinsed five times in 0.1% Tween-20 at 10 min intervals. The nuclear stain 4′,6-diamidino-2-phenylindole (DAPI) was used at 1:5000 dilution and sections were incubated for 20 min in the dark. Imaging was done using a LSM710 laser confocal microscopy (Zeiss, Germany).

### Immunohistochemistry of F/G-Actin

After different treatments, blood vessels were sliced cross-sectionally into frozen sections of 4 μm thickness and fixed in 4% formaldehyde, 0.2% Triton X-100 in the PEM cytoskeleton stabilizing buffer (100 mmol/L PIPES, 5 mmol/L EGTA, 2 mmol/L MgCl_2_, pH = 6.9) for 20 min at room temperature. They were then rinsed twice in PBS for a few seconds and permeabilized with 0.2% Triton X-100 in PBS for 15 min. Thereafter, sections were blocked with blocking solution (1% BSA and 0.1% Triton X-100 in PBS) for 10 min and washed with PBS, followed by incubation with 100 nmol/L red fluorescent F-actin stain (Actin-stain 555 phalloidin, Cytoskeleton, Denver, CO, USA) and 300 nmol/L green fluorescent G-actin stain (Deoxyribonuclease I Alexa fluor-488 conjugate, Molecular Probes, USA) in blocking buffer for 20 min at room temperature in the dark. Confocal images of F-actin and G-actin were captured simultaneously with a fluorescence microscope Zeiss LSM710 (Zeiss, Germany).

### Reactive Oxygen Species Analysis

Following treatment, aorta were cross-sectionally sliced (4 μm thickness) and then stained with DHE dye conjugated to Alexa Fluor 594 (Sigma-Aldrich, St. Louis, MO, USA) at a concentration of 10 μmol/L in *N*-HEPES buffer and placed for 30 min at 37°C, 5% CO_2_. Mounting dye containing DAPI was added to the sections to stain the nuclei. Images were acquired using LSM710 (Zeiss, Germany).

### Drugs

*S*-nitroso-*N*-acetylpenicillamine was prepared as stock solution in DMSO and diluted in the *N*-HEPES buffer solution. L-NAME, Cyt-D, APN, cGMP, cGMPS were directly dissolved in the *N*-HEPES buffer solution.

Control groups received same concentrations of vehicle*s* (diluted DMSO or *N*-HEPES buffer).

### Statistical Analysis

Values are presented as mean ± standard error of the mean (SEM). Data were analyzed using one-way analysis of variance (ANOVA) or *t*-test. Significance was established by Tukey or Holm-Sidak methods. *p* < 0.05 was considered to represent significant differences.

## Results

### The Effect of Adiponectin on Ang II-Induced Protein Synthesis is Nitric Oxide-Dependent

**FIGURE 1:**
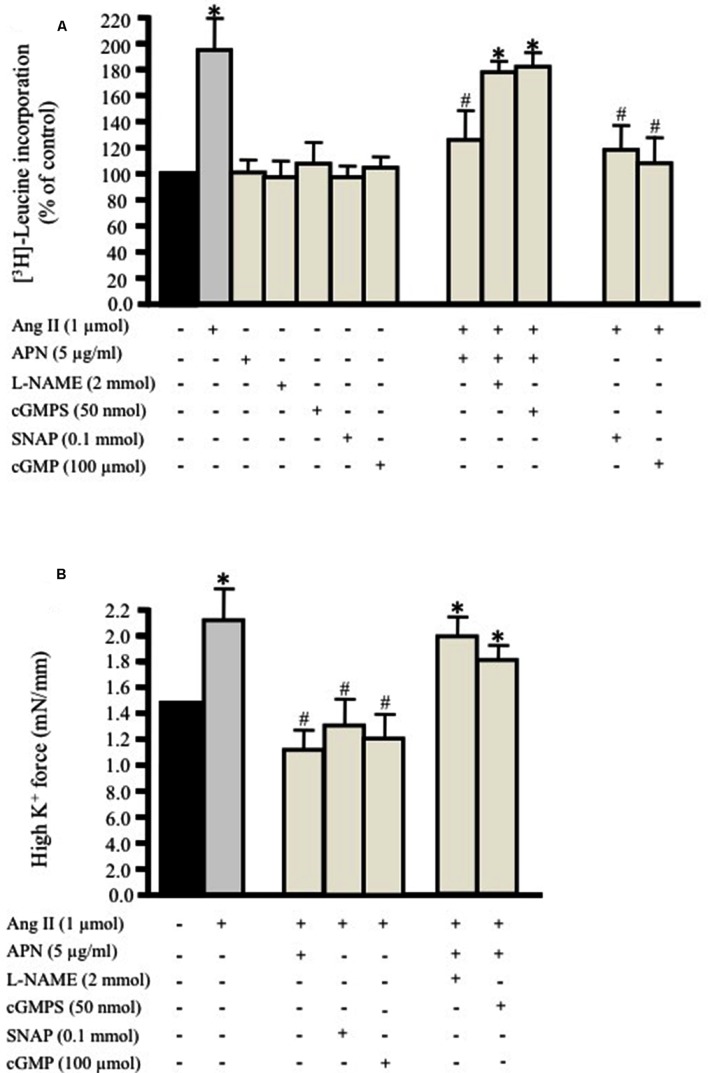
**Adiponectin inhibits Ang II-induced protein synthesis and force production in rat aortic ring.** Serum-starved endothelium-intact rat aortic rings were pre-treated with adiponectin (5 μg/ml), L-NAME (2 mmol/L), cGMPS (50 nmol/L), *S*-nitroso-*N*-acetylpenicillamine (SNAP; 0.1 mmol/L), 8-Br-cGMP (cGMP; 100 μmol/L) for 1 h prior to 24 h treatment with 1 μmol/L Ang II. [^3^H]-leucine incorporation is shown in **(A)**. Active stress in response to high K^+^ is shown in **(B)**. Data are shown as mean ± SEM with *n* = 5–6 for all groups. ^∗^*p* < 0.05 vs. without Ang II (control); #*p* < 0.05 vs. with Ang II.

We investigated whether a physiological concentration of adiponectin (5 μg/ml; Ouchi et al., 1999) had an anti-hypertrophic effect on Ang II-induced protein synthesis in VSMC. Endothelium-intact and denuded aortic rings were treated with Ang II (1 μmol/L; Coles et al., 2007) for 24 h with [^3^H]-leucine in order to study the effect of Ang II on protein synthesis. In control aortic rings, which were not exposed to Ang II, only weak protein synthesis was observed (**Figure 1A**). Both endothelium-intact and denuded aortic tissue exposed to Ang II exhibited a significant increase in protein synthesis by 190 ± 21% (**Figure 1A**) and 180 ± 16% respectively. Pre-treatment of aortic rings with adiponectin (5 μg/ml) for 1 h and then co-incubated with 1 μmol/L Ang II significantly inhibited Ang II-induced protein synthesis in endothelium-intact (127 ± 19%; **Figure 1A**) and denuded aortic tissue (118 ± 11%).

Moreover, we determined whether inhibition of either NO generation by L-NAME (2 mmol/L; Day et al., 1999) or cGMP by the specific inhibitor of cGMP-dependent protein kinase Rp-8-Br-PET-cGMPS (cGMPS, 50 nmol/L) prevented the inhibitory effect of adiponectin on Ang II-induced protein synthesis in endothelium-intact aortic rings. Both compounds strongly inhibited the anti-hypertrophic action of adiponectin (**Figure 1A**) to almost the control level. These data suggest the possible role of NO synthesis and cGMP in the anti-hypertrophic effect of adiponectin against Ang II-induced protein synthesis.

### NO/cGMP Activation Attenuates Ang II-Induced Hypertrophy

We tested the hypothesis that the NO/cGMP pathway attenuates Ang II-induced protein synthesis in the endothelium-intact aortic ring. We studied the effect of the NO-donor SNAP (0.1 mmol/L) or the cGMP analog 8-Bromo-cGMP (cGMP, 100 μmol/L; Hofbauer et al., 2002) on Ang II-induced protein synthesis in aortic rings cultured for 24 h. Pre-treating the endothelium-intact aortic rings with either the NO donor SNAP (0.1 mmol/L) or 8-Br-cGMP (100 μmol/L) significantly attenuated Ang II-induced vascular hypertrophy (**Figure 1A**). The inhibition of Ang II-induced protein synthesis caused by SNAP was reversible. Re-culturing the aortic tissue in new media containing Ang II for 24 h made the total protein synthesis return to control levels.

### Adiponectin, NO, and cGMP Decrease Vascular Contractility

We then examined the impact of pre-treating the aortic ring with adiponectin (5 μg/ml), SNAP (0.1 mmol/L), or cGMP (100 μmol/L) followed by treatment with Ang II (1 μmol/L) for 24 h on high K^+^-induced vascular contractility. The force of aortic rings was normalized to cross-sectional area in order to have active stress. **Figure 1B** shows that the aortic rings cultured for 24 h with Ang II alone had greater active stress during high-K^+^ stimulation at optimal length compared to pre-treated aortic rings with adiponectin, SNAP, or cGMP. On other hand, pre-treating the aortic ring with L-NAME (2 mmol/L) or Rp-8-Br-PET-cGMPS (cGMPS, 50 nmol/L) for 1 h followed by Ang II (1 μmol/L) for 24 h significantly decreased VSMC contractility induced by high K^+^ (**Figure 1B**).

### Adiponectin Attenuates Ang II-Induced RhoA Activation

**FIGURE 2:**
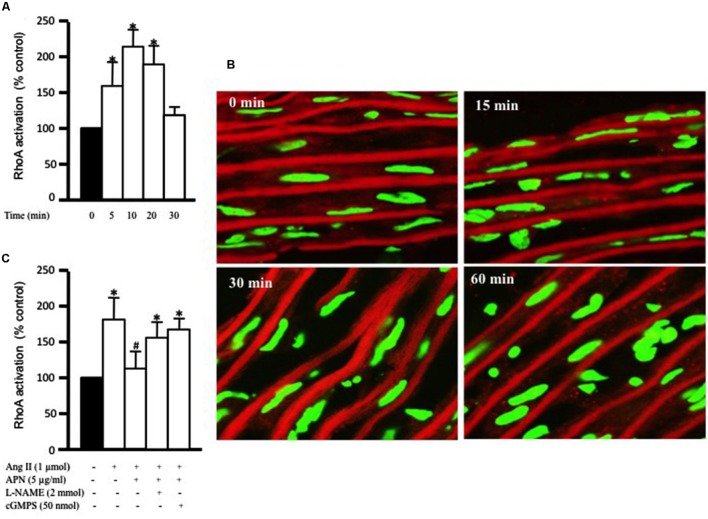
**Adiponectin inhibition of Ang II-induced RhoA activation is NO-dependent.** **(A)** Endothelium-intact rat aortic rings were treated for various times (0, 15, 30, and 60 min) with 1 μmol/L Ang II and RhoA activation was analyzed with G-LISA kit BK121. **(B)** Time course of Ang II-induced RhoA translocation. Confocal images for aortic frozen sections at different time points (0, 15, 30, 60 min) stained with DAPI (green) and anti-RhoA antibody (red). **(C)** Endothelium-intact rat aortic rings were treated for 10 min with 1 μmol/L Ang II in the presence or absence of adiponectin (5 μg/ml), L-NAME (2 mmol/L), cGMPS (50 nmol/L). RhoA activation was analyzed with G-LISA kit BK121. Data are shown as mean ± SEM and indicate fold change relative to untreated aortic ring (control). *n* = 6–7; ^∗^*p* < 0.05 vs. without Ang II.

The critical role of RhoA activation has been shown in vascular hypertrophy and contractility induced by several factors, including Ang II (Yamakawa et al., 2000; Molkentin and Dorn, 2001). Our aim was to study the effect of adiponectin on Ang-II-induced RhoA activation. **Figure 2A** shows that exposure of aortic rings to 1 μmol/L Ang II caused a time-dependent increase in RhoA activation, which was evident after 5 min and peaked at 10 min. There appeared to be a decline in RhoA activity with Ang II treatment for a long duration. To determine whether Ang II-induced RhoA activation in the aorta was associated with cytosolic RhoA translocation to membrane, we investigated RhoA translocation in aortic rings using immunohistochemistry. **Figure 2B** shows that Ang II treatment for 30 min noticeably induced RhoA translocation from the cytosol to the cell membrane, further indicating RhoA activation.

We then examined whether the anti-hypertrophic effect of adiponectin was mediated via inhibition of RhoA activation. Endothelium-intact aortic rings were pre-treated with adiponectin for 1 h followed by Ang II treatment for 10 min, the peak of Ang II-induced RhoA activation. **Figure 2C** shows that adiponectin (5 μg/ml) significantly inhibited Ang II-induced RhoA activation. The inhibitory effect of adiponectin on Ang II-induced RhoA activation was attenuated by L-NAME (2 mmol/L) and cGMPS (50 nmol/L), indicating the involvement of NO action in the inhibitory effect of adiponectin on Ang II-induced RhoA activation.

### NO Attenuates Ang II-Induced RhoA Activation

**FIGURE 3:**
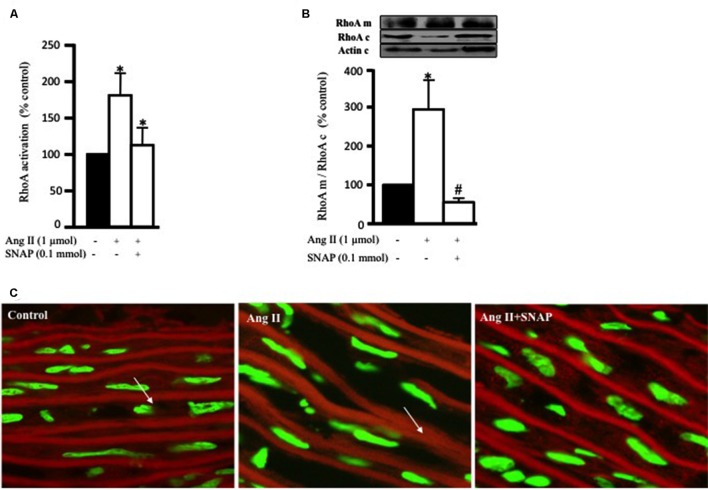
**Nitric oxide (NO) inhibits Ang II-induced RhoA activation and translocation.** **(A)** Endothelium-intact rat aortic rings were cultured for 10 min with or without 1 μmol/L Ang II pre-treated with/without SNAP and RhoA activation was analyzed with G-LISA kit BK121. **(B)** A representative Western blot showing RhoA membrane fraction (RhoA m), RhoA cytosolic fraction (RhoA c) and actin (actin c) after 30 min treatment with Ang II in aortic rings. Ang II caused a threefold increase in membrane bound RhoA whereas SNAP inhibited RhoA translocation. Each bar represents mean ± SEM value obtained from 3 to 7 independent experiments. ^∗^*p* < 0.05 vs. without Ang II. #*p* < 0.05 vs. with Ang II. **(C)** Confocal images for aortic ring frozen sections stained with DAPI (green) and anti-RhoA antibody (red) treated with Ang II for 30 min with or without SNAP. Control (left panel), Ang II (middle panel), Ang II + SNAP (right panel).

The ability of NO to inhibit RhoA activity has been studied in cardiac cells (Kuwahara et al., 1999) and VSMC (Murthy et al., 2003). In this study, we examined whether the anti-hypertrophic effect of NO was mediated via inhibition of Ang II-induced RhoA activation after 10 min of Ang II treatment in endothelium-intact aortic rings. **Figure 3A** show that SNAP (NO donor; 0.1 mmol/L) significantly inhibited Ang II-induced RhoA activation (**Figure 3A**).

In order to examine the translocation of RhoA after 30 min of Ang II treatment, we performed a Western blot analysis on cytosolic and membrane fractions of rat aortic rings. In addition, we examined whether the anti-hypertrophic effect of NO was mediated via inhibition of Ang II-induced RhoA translocation, using SNAP. Treatment with Ang II significantly increased RhoA translocation from the cytosol to the cell membrane by almost threefold as compared to controls (**Figures 3B,C**). This was assessed as the ratio of total RhoA in the membrane to that in the cytosol. As expected, SNAP significantly inhibited Ang II-induced RhoA translocation (**Figures 3B,C**).

### NO Attenuates Ang II-Induced Cofilin-2 Phosphorylation

**FIGURE 4:**
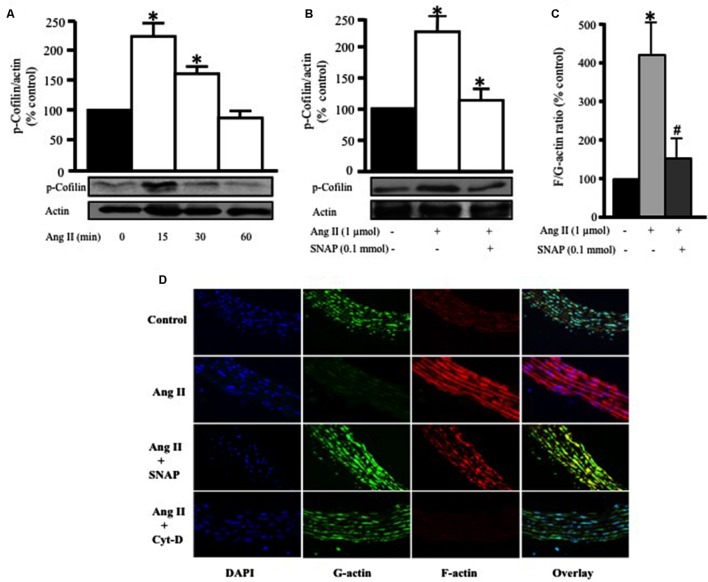
**Nitric oxide attenuates Ang II-induced RhoA/ROCK pathway activation and actin cytoskeleton remodeling.** **(A)** Endothelium-intact rat aortic rings were treated for various times (0, 15, 30, and 60 min) with 1 μmol/L Ang II and cofilin-2 phosphorylation was analyzed by Western Blotting. *n* = 5; ^∗^*p* < 0.05. **(B)** SNAP significantly inhibited Ang II-induced cofilin-2 phosphorylation after 15 min. Endothelium-intact rat aortic rings were treated with Ang II for 24 h with or without SNAP followed by quantification of F/G-actin ratio **(C)**. Confocal images of aortic ring sections stained with DAPI (blue, nucleus), Deoxyribonuclease-I Alexa Fluor 488 (green, G-actin) and F-Phalloidin-555 actin stain (red, F-actin). Rat aortas were treated for 24 h with 1 μmol/L Ang II in the presence or absence of SNAP (0.1 mmol/L) or Cyt-D (1 μmol/L) and relative amounts of F- and G-actin were assessed **(D)**. *n* = 5–7; ^∗^*p* < 0.05 vs. without Ang II; #*p* < 0.05 vs. with Ang II.

To further confirm RhoA translocation and activation as a potential mediator of Ang II-induced hypertrophy in endothelium-intact aortic rings, we assessed the phosphorylation of cofilin-2, an actin depolymerizing protein and a downstream effector of RhoA/ROCK activation. Ang II (1 μmol/L) significantly increased cofilin-2 phosphorylation, as compared to control, in a time-dependent manner, with a peak at 15 min (^∗^*p* < 0.05, **Figure 4A**). This indicates that cofilin-2 is phosphorylated and thus inhibited by Ang II-induced RhoA translocation, an indicator of RhoA/ROCK pathway activation. In order to investigate whether NO modulates Ang II-induced cofilin-2 phosphorylation, SNAP (0.1 mmol/L) was used as source of NO. **Figure 4B** shows that Ang II-induced cofilin-2 phosphorylation was significantly inhibited by NO in comparison to Ang II alone after 15 min (^∗^*p* < 0.05).

### NO Modulates Ang II-Induced Actin Cytoskeleton Remodeling

Previous studies demonstrated that actin cytoskeleton dynamics are the major target of phosphorylated cofilin-2 in the RhoA/ROCK pathway (Zeidan et al., 2006). In this study we investigated the effect of Ang II treatment for 24 h on variations of F- to G-actin ratio in endothelium-intact aortic rings. Ang II (1 μmol/L) significantly increased F/G-actin ratio by fourfold, indicating a large pool of filamentous F-actin in comparison to G-actin (^∗^*p* < 0.05, **Figure 4C**). However, this effect was completely inhibited by SNAP treatment (**Figure 4C**). Thus, Ang II-activation of RhoA indeed induces changes in actin cytoskeleton dynamics, which is inhibited by NO.

To further confirm our findings, confocal microscopy was performed (**Figure 4D**) using Phalloidin and Deoxyribonuclease I to stain F-actin (red) and G-actin (green), respectively. The nuclei of VSMC were stained with DAPI for DNA (blue). Control aortic ring sections showed more G-actin compared to F-actin (**Figure 4D**). On the other hand, Ang II-treated aortic rings for 24 h showed a significant increase in F-actin compared to G-actin (**Figure 4D**). However, F-actin was markedly decreased following SNAP (0.1 mmol/L) or Cyt-D (1 μmol/L; Ghantous et al., 2015b) treatment (**Figure 4D**). These results suggest that the protective effect of NO is mediated by actin cytoskeleton dynamics through increasing F/G-actin ratio.

### Adiponectin and NO Inhibit Ang II-Induced ROS Production

**FIGURE 5:**
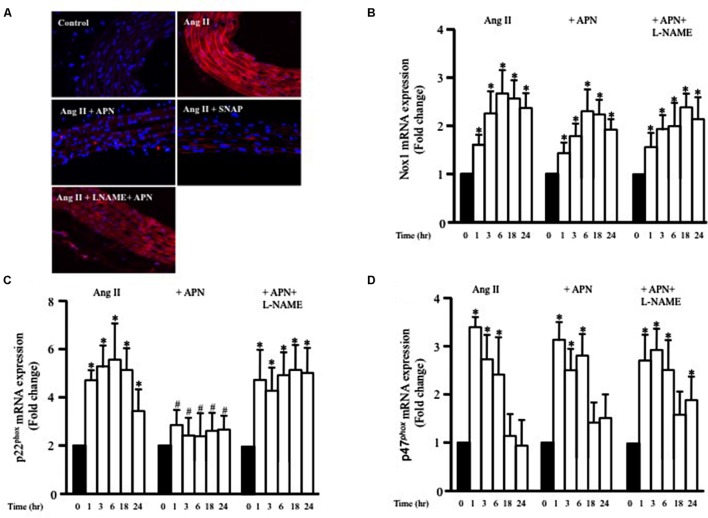
**Nitric oxide mediates the inhibitory effect of adiponectin on Ang II-induced ROS formation and p22^phox^ mRNA overexpression.** **(A)** Representative confocal microscopic images of aortic wall treated with Ang II for 1 h, with or without inhibitors and agonists, followed by staining with DHE (red). DAPI stained the nuclei blue. Fold change of Nox1 **(B)**, p22^phox^ **(C)**, and p47^phox^ **(D)** mRNA expression levels in the aortic rings after 0, 1, 3, 6, 18, 24 h of Ang II (1 μmol/L) treatment. *n* = 8–10 in each group; ^∗^*p* < 0.05 vs. control, #*p* < 0.05 vs. with Ang II.

Reactive oxygen species has been shown to be implicated in the development of vascular hypertrophy (Ghantous et al., 2015b). In order to determine whether Ang II-induced hypertrophy is associated with ROS production, endothelium-intact aortic rings were stained with DHE and viewed using a laser confocal microscope. Untreated aortic section barely generated ROS (**Figure 5A**). Ang II treatment for 1 h noticeably upregulated ROS formation in comparison to control aortas (**Figure 5A**). However, adiponectin and SNAP completely inhibited Ang II-induced ROS production (**Figure 5A**), as compared to Ang II-treated aortas. The inhibitory effect of adiponectin on Ang II-induced ROS formation (for 1 h) was abolished by L-NAME, indicating the involvement of NO action in the inhibitory effect of adiponectin on Ang II-induced ROS formation.

### Ang II-Induced p22^phox^ mRNA Expression is Inhibited by NO and cGMP

NADPH oxidase (Nox) expression and activity are critical factors for ROS formation in various cells types, including VSMC. To examine whether Ang II enhanced the expression of Nox1 mRNA, endothelium-intact aortic rings were stimulated with Ang II (1 μmol/L) for 1, 3, 6, 18, or 24 h and the mRNA expressions of Nox1 was analyzed using qPCR. **Figure 5B** shows that Ang II significantly enhanced Nox1 expression after 1 h and was further enhanced up to 24 h. We also assessed whether adiponectin (5 μg/ml) could prevent the Ang II-induced upregulation of Nox1 mRNA expression using qPCR analysis. **Figure 5B** shows that adiponectin and adiponectin + L-NAME had no effect on Ang II-induced Nox1 overexpression.

To explore further the involvement p47^phox^ and p22^phox^ on Ang II-induced ROS formation in VSMC, we investigated whether the expression of p47^phox^ and p22^phox^ mRNA in endothelium-intact aortic rings could be modified by Ang II treatment. Ang II significantly increased both p47^phox^ and p22^phox^ mRNA expression in a time-dependent manner (**Figures 5C,D**). Upregulation of p47^phox^ mRNA expression induced by Ang II was not affected when aortic rings were pre-treated with adiponectin compared with the controls (**Figure 5D**). On the other hand, adiponectin significantly decreased p22^phox^ mRNA expression compared with the Ang II-treated group (**Figure 5C**). Collectively, these data indicate the inhibitory effect of adiponectin on Ang II-induced p22^phox^ overexpression but not on Ang II-induced Nox1 or p47^phox^ overexpression.

**FIGURE 6:**
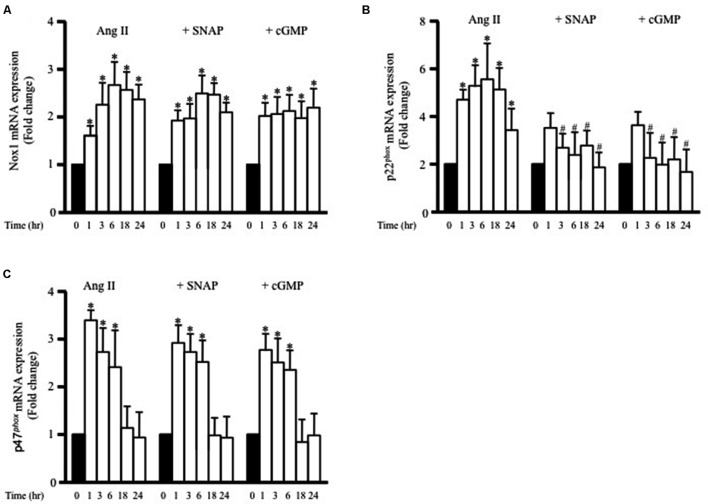
***S*-nitroso-*N*-acetylpenicillamine and cGMP attenuate Ang II-induced p22^phox^ mRNA expression.** Fold change of Nox1 **(A)**, p22^phox^ **(B)**, and p47^phox^ **(C)** mRNA expression levels in the endothelium-intact rat aortic rings after 0, 1, 3, 6, 18, 24 h of Ang II (1 μmol/L) treatment. *n* = 8–10 in each group; ^∗^*p* < 0.05 vs. control; #*p* < 0.05 vs. with Ang II.

To determine whether adiponectin inhibits Ang II-induced p22^phox^ overexpression through NO synthesis, L-NAME was used. **Figure 5C** shows L-NAME significantly attenuates the inhibitory effect of adiponectin on Ang II-induced p22^phox^ overexpression in endothelium-intact aortic rings. Next, we determined whether increased Nox1, p47^phox^, or p22^phox^ mRNA expression by Ang II was inhibited by SNAP or cGMP. Neither NO or cGMP had any effect on Ang II-induced Nox1 or p47^phox^ expression (**Figures 6A,C**). Similar to adiponectin effect, NO and cGMP significantly inhibited Ang II-induced p22^phox^ overexpression (**Figure 6B**).

## Discussion

Hypertension is a key risk factor for cardiovascular disease. Abnormal vascular structure and function, such as hypertrophy, increased constrictor, or decreased dilatory responses, are major aspects underlying vascular pathology in hypertension (Touyz, 2003). Ang II, an endogenous vasoconstrictor, contributes to these aspects mainly by inducing vascular hypertrophy (Zhang et al., 2005; Li et al., 2011). The ability of Ang II to activate both ROS production and RhoA/ROCK pathway has been previously investigated, suggesting the emergence of these pathways as critical components of the hypertrophy program (Zeidan et al., 2005; Jin et al., 2006) and as such represent possible targets for therapeutic intervention. The purpose of our study was to investigate whether the anti-hypertrophic effect of adiponectin on Ang II-induced hypertrophy is mediated by NO synthesis and due to inhibition of RhoA activation and ROS formation.

**FIGURE 7:**
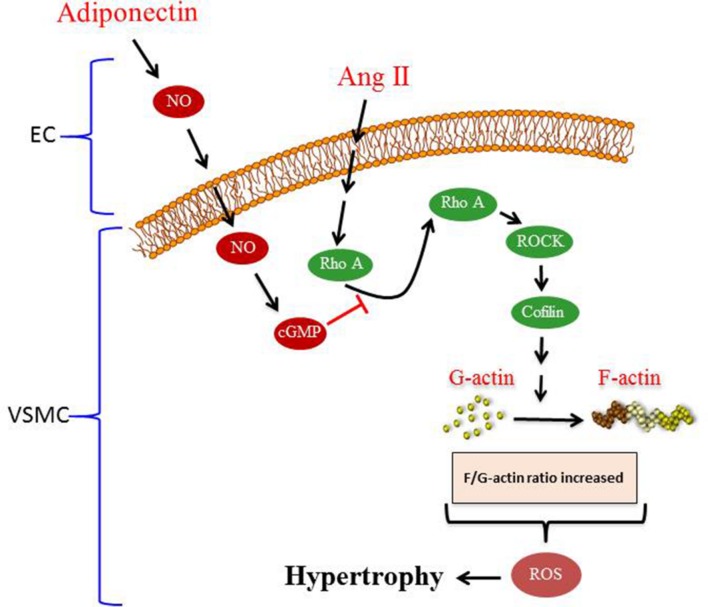
**Proposed model of adiponectin-mediated inhibition of Ang II-induced VSMC remodeling.** Treatment with Ang II induces hypertrophy in VSMC. Adiponectin suppresses AngII-induced VSMC hypertrophy through signaling pathways that activates NO synthesis in endothelial cells (ECs). The inhibitory effect of adiponectin on Ang II-induced VSMC hypertrophy is mediated through modulating the RhoA/ROCK signaling pathway, F-actin to G-actin ratio, and ROS formation.

To our knowledge, the major novel findings in our study are (1) adiponectin attenuates Ang II-induced VSMC hypertrophy, (2) adiponectin displays anti-oxidant effects and suppresses the expression of p22^phox^ mRNA expression, but not Nox1 and p47^phox^ mRNA expression in Ang II-stimulated aortic rings, and (3) the anti-hypertrophic and anti-oxidant effects of adiponectin are mediated by NO synthesis and action (**Figure 7**).

Recent studies portrayed adiponectin as a cardioprotective hormone in obesity-related diseases, such as hypertrophic cardiomyopathy, and its ability to maintain the cardiovascular function (Ouchi et al., 2006; Kitaoka et al., 2010). Indeed, in contrast to other adipokines, circulating APN levels are reduced in cardiovascular diseases (Shetty et al., 2009). The effects of APN occur through interaction with specific APN receptors, AdipoR1 and AdipoR2. These receptors are expressed in several cells including VSMC (reviewed by Ghantous et al., 2015a). Upon binding to its receptors, adiponectin activates several signal transduction pathways mediated by AMPK and PPARα (Yamauchi and Kadowaki, 2013). Through activation of AMP-activated protein kinase signaling, adiponectin has been demonstrated to inhibit the hypertrophic effect of Ang II on cardiac tissue (Wang et al., 2010). Adiponectin-deficient mice exposed to pressure overload showed increased cardiac hypertrophy, while supplementation of adiponectin protected against the development of cardiac hypertrophy in response to angiotensin II (Shibata et al., 2004). To our knowledge, there aren’t studies done on angiotensin II-infusion in adiponectin receptor-deficient mice. However, angiotensin II-infusion done on normal rats induced cardiac hypertrophy and reduced the expression of AdipoR1 but not AdipoR2 (Li et al., 2013). Angiotensin II-infusion also reduced circulating levels of adiponectin (Li et al., 2013).

To date and to our knowledge, there are no studies that have examined the anti-hypertrophic effect of adiponectin in Ang II-induced vascular hypertrophy. In this study, we approached this question and we showed the anti-hypertrophic effect of adiponectin on Ang II-induced vascular hypertrophy. The next step was to understand the molecular mechanism of the anti-hypertrophic effect of adiponectin. Increasing attention has recently been focused on the role of ROS in adiponectin signal transduction (Fujimoto et al., 2010; Yuan et al., 2012). Accumulating evidence suggests that ROS is involved in the hypertrophic responses to Ang II (Schiffrin and Touyz, 2003; Dai et al., 2011). ROS mediates VSMC hypertrophy through activation of different signaling pathways such as ERK1/2, different transcription factors, and ion channels. NADPH oxidase (Nox) generates superoxides that affect different cellular functions (Hingtgen et al., 2006). Nox1, an isoform of Nox family, exists in VSMC and participates in many vascular functions, such as muscle contraction (Matsuno et al., 2005). Nox1 is composed of several subunits including p22^phox^ and p47^phox^. Both p22^phox^ and p47^phox^ are necessary for Nox1 function and stability (Lambeth, 2004), which leads to ROS formation. Additionally, several studies have shown that Ang II induces ROS formation through upregulation of different NADPH oxidase subunits, including Nox1, p47^phox^, and p22^phox^ in cardiac and vascular cells (Touyz et al., 2005; Thomas et al., 2006). Our results in this study show, in the first place, that adiponectin has the ability to inhibit Ang II-induced ROS formation and p22^phox^ mRNA expression. Interestingly, treatment with adiponectin failed to inhibit the expression of both Nox1 and p47^phox^. As far as we know, this is the first report of the protective effect of adiponectin on ROS and downregulation of p22^phox^ mRNA expression.

To evaluate the molecular mechanisms underlying the inhibitory effect of adiponectin on ROS formation and p22^phox^ mRNA expression in Ang II-stimulated aortic rings, we investigated the influence of the NO synthesis inhibitor L-NAME on adiponectin’s anti-oxidant effect. NO has many cardioprotective actions on the cardiovascular system during hypertension (Tanaka and Node, 2015). The major functions of NO on the cardiovascular system include anti-inflammatory, anti-proliferative, anti-thrombotic, and anti-hypertrophic activity (Tziros and Freedman, 2006; Garcia and Incerpi, 2008; Momi et al., 2012). However, the exact molecular mechanisms of the anti-hypertrophic effect of NO on Ang II-induced VSMC hypertrophy remain unclear. VSMC contractility is associated with hypertrophy when exposed to high stress such as hypertension. We and others have shown that Ang II significantly increased the VSMC force-generating ability in response to high-K^+^ depolarization (Garcha et al., 2001). Different studies have shown the involvement of the RhoA/ROCK pathway, which is activated by Ang II (Yamakawa et al., 2000), in VSMC contraction (Sakamoto et al., 2003; Zeidan et al., 2003). The observed increase in force generation was associated with increased fiber stress formation (increased F-actin to G-actin ratio). Indeed, actin cytoskeleton remodeling and stress formation are a well-described consequence of RhoA/ROCK pathway activation (Zeidan et al., 2007). It is reasonable to speculate that actin cytoskeleton modulation would increase the contractile potential of VSMC. Another potential factor that leads to increased force production is myosin light chain phosphatase (MLCP). Inhibition of MLCP by RhoA/ROCK pathway has been shown to increase calcium sensitivity, leading to an increase in muscle contraction (Mizuno et al., 2008).

In this study we have investigated the role of Rho A activation and translocation in Ang II -induced VSMC remodeling. To activate the RhoA/ROCK pathway, RhoA must first be activated by binding to GTP [by guanine nucleotide exchange factors (GEFs)], then translocated to the cell membrane, followed by subsequent activation of ROCK and the pathway (Zeidan et al., 2007). We have shown that Ang II treatment caused a significant activation in RhoA after 5 min, which peaked after 10 min (**Figure 2A**). We then examined whether RhoA activation was followed by its translocation to the membrane. Ang II treatment for 15 min showed some RhoA translocation to the membrane, while 30 min of Ang II treatment induced a marked increase in RhoA translocation (**Figure 2B**). Hence, RhoA activation after 10 min of Ang II treatment was indeed followed by its translocation to the membrane, which began after 15 min and peaked after 30 min of Ang II treatment. Since changes in F-actin and G-actin cytoskeleton dynamics are the downstream targets of RhoA/ROCK pathway, their levels were examined. Indeed, time-course experiments for F- and G-actin cytoskeleton dynamics (unpublished data) showed that the changes are most prominent after 24 h, in both VSMC and cardiomyocytes (Zeidan et al., 2006, 2007, 2008; Ghantous et al., 2015b).

Nitric oxide is a gaseous molecule that acts as a vasodilating factor in VSMC while it’s an anti-hypertrophic factor in both VSMC and cardiomyocytes (Hunter et al., 2009; Van Hove et al., 2009). It diffuses easily to VSMC, across the plasma membrane, and causes their relaxation by reducing intracellular calcium levels (Van Hove et al., 2009). NO attenuates VSMC contraction and hypertrophy by inhibiting ROCK in the RhoA/ROCK pathway (Maruhashi et al., 2014). The anti-hypertrophic effect of NO is associated with vasodilatory actions though cGMP formation and phosphorylation of PKG (Kapakos et al., 2010). Our data show that culturing endothelium-intact aortic rings either with NO or adiponectin in presence of Ang II for 24 h significantly decreased force production of aorta in response to high-K^+^ depolarization compared to tissues cultured with Ang II alone. Inhibition of NO synthesis by L-NAME or cGMP-dependent protein kinase by Rp-8-r-PET-cGMPS (cGMPS) ameliorated adiponectin’s effect on Ang II-induced force production and ROS formation, indicating the necessity of NO/cGMP pathway for the inhibitory effect of adiponectin against Ang II-induced increased in muscle force production, and ROS formation. This data provide insight into how this circulating adipokine might attenuate Ang II-induced vasoconstriction and ROS formation through NO production.

To further explore another possible mechanism of the anti-hypertrophic effect of adiponectin, we attempted to investigate the involvement of the RhoA/ROCK pathway. Indeed, numerous studies have implicated the potential involvement of RhoA activation and increased stress fiber formation with the hypertrophic effect of Ang II (Yamakawa et al., 2000; Jin et al., 2006). Upon stimulation, RhoA activates a signaling cascade involving ROCK, LIMK, and cofilin-2, an actin depolymerizing enzyme that binds to actin and prevents stress fiber formation (Zeidan et al., 2007). As such, when cofilin-2 is phosphorylated (inactivated) by LIMK, the F/G-actin equilibrium is shifted toward enhanced stress fiber formation or more F-actin formation (Zeidan et al., 2008). Consistent with the previously reported findings (Hunter et al., 2009; Zeidan et al., 2014), our results also demonstrate that Ang II-induced RhoA activation and translocation are associated with cofilin-2 phosphorylation and enhanced stress fiber formation, which were abolished by treatment with NO synthesis. The effect of Ang II on cofilin-2 phosphorylation and actin cytoskeleton remodeling was abolished in aortic tissue treated with NO donor, indicating that the anti-hypertrophic effect of NO may be mediated via inhibition of RhoA activation and its downstream signaling. In addition, we showed that the ability of adiponectin to modulate the effect of Ang II on RhoA activation and actin cytoskeleton dynamics was mediated through NO synthesis.

Overall, our study provides insight into the signal transduction pathway mediating the anti-hypertrophic properties of adiponectin during hypertension. The proposed mechanism underlying the anti-hypertrophic effect of adiponectin on Ang II-induced hypertrophy is shown in **Figure 7**. Our study highlights the effects of adiponectin on cellular NO synthesis, ROS generation, RhoA/ROCK pathway, and actin cytoskeleton dynamics. Our findings could be useful in the development of novel adiponectin-targeted therapeutic interventions for the treatment of vascular hypertrophy during hypertension.

## Author Contributions

WN-E, CMG, LD, HI, and AZ contributed in generating experimental data. CMG, NE-Z, Hana A. Itani, KZ, and AZ contributed in discussion and reviewed/edited manuscript. CMG, KZ, and AZ wrote the manuscript and drew the figures.

## Conflict of Interest Statement

The authors declare that the research was conducted in the absence of any commercial or financial relationships that could be construed as a potential conflict of interest.
